# Bayesian Federated Inference for regression models based on non-shared medical center data

**DOI:** 10.1017/rsm.2025.6

**Published:** 2025-03-10

**Authors:** Marianne A. Jonker, Hassan Pazira, Anthony C. C. Coolen

**Affiliations:** 1 Research Institute for Medical Innovation, Science Department IQ Health, Section Biostatistics, Radboud University Medical Center, Nijmegen, Netherlands; 2 DCN Donders Institute, Faculty of Science, Radboud University, Nijmegen, Netherlands; 3 Saddle Point Science Europe, Mercator Science Park, Nijmegen, Netherlands

**Keywords:** data integration, decentralized data, distributed inference, Federated Learning, one-shot algorithm

## Abstract

To estimate accurately the parameters of a regression model, the sample size must be large enough relative to the number of possible predictors for the model. In practice, sufficient data is often lacking, which can lead to overfitting of the model and, as a consequence, unreliable predictions of the outcome of new patients. Pooling data from different data sets collected in different (medical) centers would alleviate this problem, but is often not feasible due to privacy regulation or logistic problems. An alternative route would be to analyze the local data in the centers separately and combine the statistical inference results with the Bayesian Federated Inference (BFI) methodology. The aim of this approach is to compute from the inference results in separate centers what would have been found if the statistical analysis was performed on the combined data. We explain the methodology under homogeneity and heterogeneity across the populations in the separate centers, and give real life examples for better understanding. Excellent performance of the proposed methodology is shown. An R-package to do all the calculations has been developed and is illustrated in this article. The mathematical details are given in the Appendix.

## Highlights

**What is already known**

- Statistical models that are estimated based on small data sets, are very likely to suffer from overfitting.
- If multiple data sets cannot be combined into one data set, the statistical analysis could be performed in a federated manner.

**What is new**

- This article describes a method for performing Bayesian federated inference (BFI) for homogeneous and heterogeneous multicenter data. In each center, the data is analyzed only once. The local inference results are centrally combined to obtain the parameter estimates without any need for repeated “cycling” across centers.
- An R software package implementing the proposed methodology is available and a manual is described in the article.

**Potential impact for RSM readers outside the authors’ field**

- The proposed methodology can be applied if data sets cannot be combined, also if the data are not of a medical nature.
- The BFI estimates are more accurate than the estimates obtained from a single center analysis.

## 1 Introduction

Prediction models aim to predict the outcome of interest for individuals (or subjects), based on their values of the covariates in the model. To build a prediction model by selecting covariates and estimating the corresponding regression parameters, the sample size should be sufficiently large. If too many variables (possible covariates) relative to the number of events or observations are included, the model may become overly flexible and erroneously ‘explain’ noise or random variations in the data, rather than estimating meaningful relationships between the covariates and the outcome. This is called overfitting and may lead to unreliable predictions of the outcome for new individuals.^1^ To overcome overfitting a minimum of 10 observations or events per variable (EPV) is often advised.^2^ ^,^ ^3^ Based on this criterion, data sets are often too small to take all available variables in consideration. Merging different data sets from different (medical) centers could in principle alleviate the problem, but is often difficult for regulatory and logistic reasons. An alternative route would be to analyse the local data in the centers and combine the obtained inference results intelligently. With this approach the (individual) data do not need to be shared across centers. In this article, we focus on methodology to combine the local inference results for estimating parametric regression models for a general population of interest. The data sets in the centers are considered as samples from this population.

In literature, several methods have been described. Probably the best-known strategy to obtain effect estimates from different inference results, is meta-analysis.^4^ In a meta-analysis, relevant, already published results are combined. Here we consider the situation where the local analyses have yet to be performed. This means that the collaborating centers discuss in advance which local analyses will be performed and what inference results should be shared to build the final combined model. It also means that more information can be shared than is usually available in publications, like the estimated covariance matrix of the estimators of the model parameters.

Federated Learning (FL) is a machine learning approach that was developed several years ago, mainly for analyzing data from different mobile devices.^5^ It aims to construct from the inference results obtained in the separate centers, what would have been found if the analysis was performed on the combined data set. With this approach, the local data stay at their owners’ centers, only parameter estimates are cycled around and updated based on the local data until a convergence criterion is met. In recent years the FL approach has improved quite a bit (e.g., on optimization in the local centers and the aggregation of the local results, dealing with heterogeneity and client-drift,^6^ ^,^ ^7^ ^,^ ^8^ ^,^ ^9^ methodology for causality related research questions^10^ ^,^ ^11^). Also FL in a Bayesian setting for deep learning models has been proposed.^12^ ^,^ ^13^ ^,^ ^14^ ^,^ ^15^ The posterior distributions are estimated in the local centers and communicated to the central server for aggregation. However, practically this Bayesian procedure is challenging, especially for deep learning models due to the high dimensionality of the parameters. An overview of the most important recent developments and a list of references is given in Liu et al.^16^ FL performs excellently in e.g., image analysis^17^ ^,^ ^18^ ^,^ ^19^ or for data from mobile devices, but has clearly some drawbacks in other applications. For instance, apart from obvious ones such as data security and convergence problems, if one aims to estimate statistical models based on inference results from different medical centers, one needs to handle challenges like heterogeneity of the populations across centers, clustering of centers, center-specific covariates (like location), missing covariates in the data, and the fact that data may be stored in different ways (covariates are named differently or are even defined differently). Furthermore, most FL strategies require many iterative inference cycles across the local centers. In case the centers are hospitals (the situation we are considering here), a cycling mechanism is complex and may lead to considerable extra work; a one-shot approach is preferred.

Also in the field of distributed statistical inference, multiple strategies have been proposed to combine inference results from different computers (centers).^20^ To cope with massive data sets which can not be analyzed on a single computer, a data set is divided into smaller data sets, which are analyzed separately and the results are combined afterwards. An interesting one-shot algorithm has been proposed by Jordan et al.^21^ They proposed a communication-efficient surrogate likelihood framework for distributed statistical inference for homogeneous data. Instead of maximizing the full likelihood for regular parametric models or the penalized likelihood in high-dimensional models, this surrogate likelihood is maximized. The surrogate likelihood expression was determined so that only a minimum amount of information is transferred from the local machines to the central server (of the order 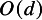 bits where *d* is the dimension of the parameter space). Later, the method was generalized to be able to deal with certain forms of heterogeneity.^22^

In this article we describe the BFI framework for parametric regression models. This methodology was developed especially for combining inference results from different centers to estimate statistical (regression) models without the need for repeated communication rounds with the local centers. In every center the data are analysed only once and the inference results (parameter and accuracy estimates) are sent to a central server, where the local inference results are combined. Explicit expressions for the combined (BFI) estimators in terms of the local inference results have been derived. Via these expressions the BFI estimates can be easily updated at a later moment if the data collection or the analysis in several centers are delayed, without contacting all other centers again (this would not be possible when using an iterative updating mechanism). The fact that only one communication round is sufficient is important in our (medical) setting, since assistance from the local medical and technical staff are needed every time local analyses are performed.

The BFI estimates are defined as the maximizers of a surrogate expression of the full log posterior density. This expression depends on the local estimates and is different from the one proposed by Jordan (2018).^21^ In the BFI framework more information (of the order 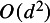 ) is shared with the central server than would normally be acceptable in a FL or distributed statistical inference setting. This additional information improves the accuracy of the estimator. The BFI methodology was developed for estimating (low-dimensional) GLMs. High dimensional models (with large *d*), typically the models of interest in FL and distributed statistical inference, are not the focus of the BFI methodology; estimation accuracy is more important than communication efficiency.

The mathematical theory of the BFI methodology for parameteric models, like GLMs, was published by the authors in Jonker et al.^23^ In this article, we extend the theory further to allow for different kinds of heterogeneity between the centers. Among others, we consider the situation in which there is heterogeneity in the population characteristics, there is clustering, the distribution of the outcome variable is shifted, and the regression or nuisance parameters differ between the centers. The asymptotic distributions of the BFI estimators are derived and it is proven that the estimators are asymptotically efficient. Asymptotically, no information is lost if the data from the centers cannot be combined. These asymptotic distributions of the estimators are used for the construction of credible intervals. For finite samples (by means of simulation studies) and asymptotically, the BFI estimators are compared to the estimators that are obtained by averaging the local estimators (weighted for local sample size). In this article, we also focus on applications: a data example is given and the R code (from our R package BFI^24^) for analyzing the data with the BFI methodology is explained.

This article is organized as follows. In Section 2 the BFI framework for generalized linear models for homogeneous sub-populations in the local centers is explained. In Section 3 different types of heterogeneity across these sub-populations and data sets are described and, moreover, it is explained how the BFI methodology can be adjusted to takes these into account. To study the performance of the BFI method in different settings, the results of simulation studies are described in Section 4. In the same section also the analysis of a heterogeneous data set using the BFI methodology is described. A discussion is given in Section 5. The article ends with three appendices. In the first appendix we explain how to do the analysis with our R package, the second appendix contains the mathematical details of the derivation of the estimators and in the third appendix the asymptotic distributions of the BFI and the weighted average estimators are derived and compared.

## 2 The Bayesian Federated Inference (BFI) framework

Suppose that data of *L* medical centers are locally available, but these data sets cannot be merged to a single integrated data set for statistical analysis. The data for individual *i* from center 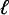 is denoted as the pair 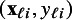 with 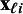 a vector of covariates and 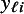 the outcome of interest. Let 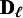 denote the data subset in center 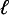 : 

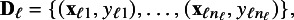

where 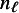 denotes the number of individuals in subset 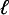 , 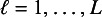 , and let 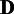 be the fictive combined data set (the union of the subsets 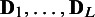 ).

The data pair 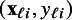 is the realisation of the stochastic pair 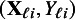 . Suppose that the variables 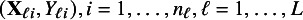 are independent and identically distributed, and that 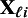 and 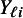 are linked via a generalized linear model (GLM) with link function *h*: 

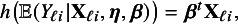

where 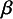 is a vector of unknown regression parameters and 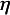 a vector of unknown nuisance parameters. If the first element in the covariate vector 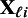 equals one for all individuals, the model includes an intercept.^i^

For 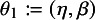 , the conditional density of 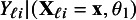 is given by 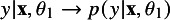 and for the vector of covariates 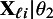 this is 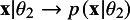 , for 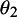 a parameter vector.^ii^ Then, for 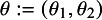 it follows that the density of 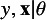 can be written as 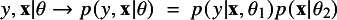 . We work in a Bayesian setting; 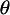 is stochastic as well. For mathematical simplicity, we assume statistical independence between 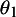 and 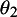 . Thus, 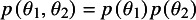 in the combined data set 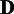 and 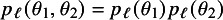 in center 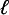 , for all 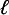 (the “ 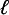 ” in the subscript refers to the center). We choose the prior parameter distributions for 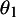 and 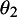 to be Gaussian with mean zero and inverse covariance matrices 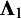 and 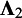 , respectively, in the combined data set, and 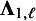 and 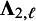 in center 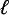 , 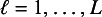 . For parameters that are positive by definition, like the variance of the error term in the linear regression model, a mean zero Gaussian prior is assumed for a transformation (e.g., the logarithm) of the parameter.

The maximum a posteriori (MAP) estimate of 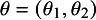 maximizes the a posteriori density of the data with respect to 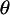 , by definition. For the combined data set 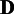 , this estimate is denoted as 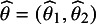 and, for the local data set 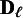 the notation 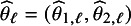 is used. If the prior density is chosen to be non-informative (large prior variances), the MAP estimates will be close to the maximum likelihood estimates. The estimator 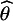 is fictive as the data set 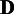 can not be created. In the following we derive expressions for 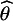 in terms of the MAP estimators based on the local data sets 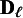 . Once the estimates in the separate centers have been found, these expressions tell us how to combine them to obtain (an approximation of) 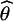 .

For the fictive combined data set 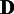 the log posterior density can be written as 
(1)

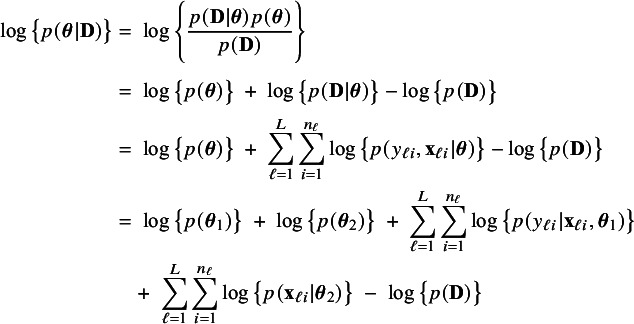

by Bayes’ rule (first equality), independence between the observations (third equality), and, among others, independence between 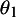 and 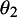 (fourth equality). Similarly, the logarithm of the posterior density in center 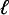 can be written as 
(2)

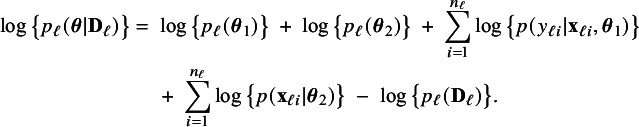

The log posterior densities 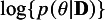 and 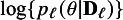 are decomposed into terms that depend on either 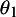 or on 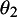 , but never on both. As a consequence, maximization with respect to 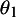 and 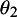 to obtain their MAP estimators can be performed independently. By reordering the terms in expression (2), we find 

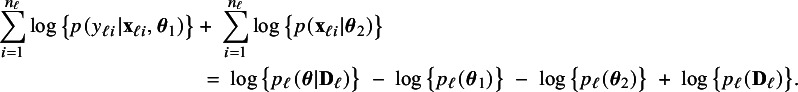

The right hand side of this expression can be inserted into expression (1). Then, the log posterior density for the full data set 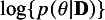 is written as a sum of the local log posterior densities in the centers and the log prior densities (more details are given in Appendix II.A). For deriving the BFI estimators of the parameters, the local log posterior densities are approximated by second order Taylor expansions around the local MAP estimates. Instead of maximizing the full log posterior density for the combined data, the quadratic approximation is maximized with respect to the parameters. The parameter value where the maximum is attained is defined as the BFI estimate. For 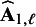 and 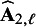 the second derivatives of 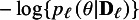 with respect to 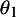 and 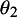 and evaluated in the local MAP estimators 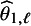 and 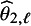 , in center 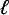 , the BFI estimators equal 
(3)

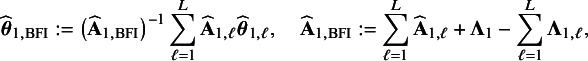



(4)

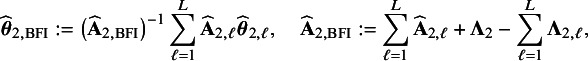

see Appendix II.A for the derivation. With these expressions we can compute approximations of 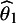 and 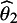 *a posteriori* from the inference results on the subsets and there is no need to do inference on the (fictive) combined data set 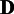 to find the BFI estimates. In the calculations of the BFI estimators, we assume independence between the parameters 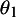 and 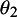 . This assumption was made for mathematical convenience, as the log posterior density splits into terms that are a function of 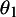 or of 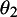 , but never of both, and as a consequence, separate expressions for 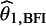 and 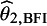 are found. This independence assumption is not essential. If the parameters are dependent, the calculations can be performed in a similar way and a single expression for the BFI estimator for 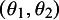 is found.

In Appendix III.B we prove that under the assumption of no model misspecification (including homogeneity between the centers), the BFI estimators 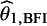 and 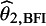 are asymptotically Gaussian and efficient (minimum asymptotic variance). For 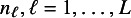 the local sample sizes and 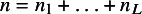 the total sample size, it is proven that 

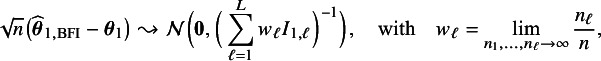

and 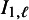 the Fisher information matrix in center 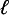 (the notation ‘ 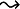 ’ means convergence in distribution). The matrix 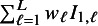 equals the Fisher information matrix for estimating 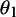 in the combined data set (see Appendix III.A). The BFI estimator asymptotically follows the same distribution as the MAP and the Maximum Likelihood estimators on the combined data. Apparently, no information is lost as a consequence of the fact that the data sets cannot be shared. In the homogeneous setting 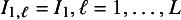 , independent of 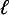 , and 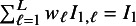 . Further, since 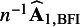 converges in probability to 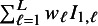 (see Appendix III.B), the asymptotic covariance matrix can be estimated by the inverse of 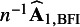 . Similar results hold for the BFI estimator 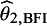 .

It follows that for a sufficiently large total sample size, the BFI estimators 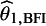 and 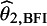 are approximately Gaussian with mean 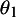 and 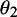 and with covariance matrices that can be estimated by 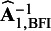 and 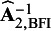 . From this, credible intervals for 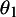 and 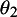 can be constructed. Let 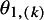 be the 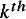 element of 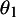 . This parameter is estimated by 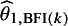 , the 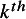 element of 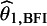 and its approximate 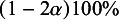 credible interval equals 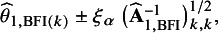 for 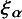 the upper 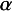 -quantile of the standard Gaussian distribution and 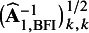 equal to the square root of the 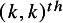 element of the inverse of the estimator 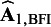 . Hypothesis testing is also straightforward by the asymptotic normality.

## 3 Heterogeneity across centers

In the derivation of the estimators for the aggregated BFI model in (3) and (4), homogeneity of the populations across the different centers is assumed. This assumption means that the parameters 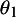 and 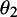 are the same in every center. This assumption may not be true, and the BFI approach has to be adjusted to take this heterogeneity into account. This is the topic of the present section.

In order to explain different types of heterogeneity, a specific example is used throughout the article. This example is also used in Section 4 and Appendix I to illustrate the BFI methodology and to study its performance. Here we give only a brief description, a more extensive description is given in Section 4.2. The example data come from a hypothetical study on stress among nurses on different wards in different hospitals.^26^ The data were simulated from a linear mixed effects model. The outcome of interest is job-related stress. For every nurse, information on stress, age, experience (in years), gender, wardtype (general, special care), hospital, and hospital size (small, medium, large) is available.

Heterogeneity in the populations across multiple centers may occur if, for instance, some medical centers are located in large cities and others in more rural areas. It might also be that in some hospitals the stress level among nurses is significantly higher than in others due to factors that are not nurse specific, like the size of the hospital or management decisions within a hospital (which are not in the data). In this section the following types of heterogeneity are considered:

1. Heterogeneity of population characteristics in the centers, e.g., the age distributions of the nurses differ. Then, the values of the parameter 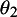 differ across centers. This is considered in Section 3.1.
2. Heterogeneity across centers in outcome mean. This may happen if the mean stress-level of the nurses vary across the centers due to factors that have not been measured (e.g., type of management). This is considered in Section 3.2.
3. Heterogeneity across centers due to interaction effects; the effect of a covariate varies across the centers. For instance, it might be that the effect of the wardtype on the outcome differs across medical centers. This means that the regression coefficient for wardtype is center-specific. This situation is considered in Section 3.3.
4. Heterogeneity across centers due to center-specific nuisance parameters; e.g., the variance of the error term in a linear regression model. See Section 3.4.
5. Heterogeneity across centers due to clustering; e.g., clustering by the location of the hospitals. This situation is considered in Section 3.5.
6. Heterogeneity across centers due to center-specific covariates. An example of such a covariate is hospital size, which is the same for every nurse in a hospital, but may vary across hospitals. See Section 3.6

These types of between-center heterogeneity are due to center-specific parameters (types 1–4), due to clustering (type 5) and due to missing covariates (type 6). There may be more forms of heterogeneity that can be taken into account with the BFI methodology. The aim of the BFI approach is to increase the sample size relative to the parameter dimension to overcome overfitting. By significantly increasing the number of parameters in the BFI model, to account for heterogeneity, the very objective of the BFI approach would thereby be undermined.

### 3.1 Heterogeneity of population characteristics

Characteristics of the populations who visit the *L* centers may differ, for instance because the centers are located in different countries or regions. In the example, the fractions of female nurses differ across the centers.

The parameter 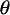 was decomposed in 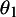 and 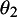 . The parameter 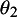 describes the distribution of the covariates 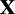 , whereas the parameter 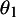 describes the relationship between the covariates and the outcome (so the regression coefficients and the nuisance model parameters). Under the assumption that 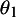 and 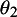 are independent, the local log posterior densities were decomposed into terms that depend on either 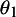 or 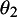 , but never on both (see expression (2)). As a consequence, when calculating the MAP estimates of 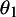 and 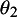 , separate functions have to be maximized. Therefore, even if we would take into account that the populations vary across the centers, the expressions of the BFI estimators 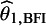 and 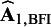 in (3) would not change and 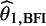 is still asymptotically unbiased. However, because the estimators depend on (summary statistics) of the covariates, the estimates 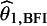 and in particularly its accuracy, which is represented by 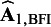 , may and often do change. This is investigated in the next section using simulation studies. For 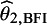 in (4) new expressions can be derived that take the heterogeneity into account. The exact expressions depend on the simultaneous distributions of the covariates and the type of heterogeneity that is assumed. Therefore, it is not possible to provide new, explicit expressions that are universally valid. In the simplest case, the covariates are assumed to be independent (which is usually not the case in practice). Then, if it is also assumed that the priors of the coordinates of 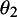 are independent, the part of the log-likelihood function that is related to the parameter 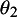 can be written as a sum of terms, where the distribution parameters corresponding to the covariates are present in distinct terms. Now new expressions for the BFI estimators of the coordinates of 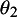 and therefore also for the vector 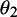 can be calculated along the same lines as in the Appendices II.B and II.C.

### 3.2 Heterogeneity across outcome means

If the combined data would be available for analysis, a multi-level model that includes a random center effect for possible unmeasured heterogeneity across centers would be considered. As an alternative one could include a fixed effect for the different centers. In both cases, this means that every center has its own center-specific intercept. At a local level, so within a center, it is not possible to estimate a center-effect. When combining the MAP estimators from the different centers into a BFI estimator for the combined model, different intercepts across the centers can be allowed in the model. This is explained below and the mathematical derivation can be found in Appendix II.B.

Suppose a regression model is fitted in every center based on the local data only. The BFI strategy as explained before, combines the fitted models to a model with a single general intercept. In Appendix II.B the BFI calculations are given for combining the local models in the situation that one or multiple regression parameters may vary across the centers and center-specific parameters are adopted in the aggregated BFI model. By taking this “varying regression parameter” to be the intercept in the resulting combined BFI model, every center has its own estimated intercept (and there is no general intercept). To be more specific, an estimate of the following aggregated BFI generalized linear model is obtained for an individual in center 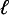



(5)

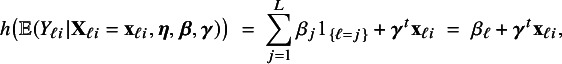

where the indicator function 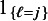 equals 1 if 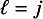 and 0 if 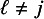 . The parameters 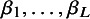 are the center-specific intercepts and 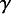 is the vector of regression parameters. The vector of covariates 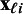 does not include a 1 for the intercept. So, the aggregated BFI model for a nurse from center 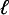 has an intercept 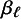 , which is specific for that center. The model can be easily rewritten into a form with a general intercept and parameters for the effect relative to the reference center which is taken to be center 1: 

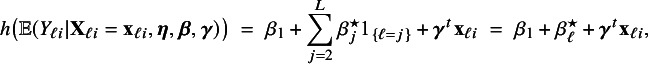

where 
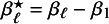
 , for 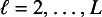 , with 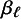 as in model (5). So, by allowing different intercepts when combining the fitted local models, the BFI model accounts for a “center-effect”.

### 3.3 Heterogeneity due to center interaction effects

Next suppose that the effect of a covariate (a regression parameter) may vary across the centers. For instance, suppose that the effect of wardtype on job related stress may differ across the centers. In the regression model for the combined data, an interaction between the covariate wardtype and the hospital would be included. To obtain these estimates with the BFI approach, the calculations from Appendix II.B can be followed again, but this time for a regression parameter instead of the intercept. That gives an aggregated BFI model of the form: 

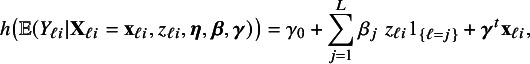

where 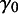 is the intercept, 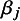 the wardtype effect on stress in center *j*, 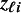 the indicator function that indicates whether nurse *i* from hospital 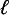 is from a special care ward (0 general, 1 special care), 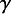 the remaining regression parameters and 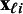 the vector of covariates (so without wardtype).

### 3.4 Heterogeneity due to having distinct nuisance parameters

The nuisance parameter of the statistical model, for example the variance of the error term in a linear regression model, may differ between the medical centers. Here too, the calculations for the BFI estimator in Appendix II.B can be applied. This yields an estimated aggregated BFI model with a specific nuisance parameter for each center.

### 3.5 Heterogeneity due to center-clustering

Local centers can be clustered based on, for example, geospatial regions, type of center (e.g., academic/non-academic hospital) or its size (small/medium/large). If the data can be combined, clustering can be taken into account by including a categorical variable in the model that represents this clustering. Within a center, this is not possible, because all persons in the center are in the same cluster and thus have the same variable value (which would lead to collinearity with the intercept); the regression model must be fitted without the corresponding variable. In this local model, the estimated intercept includes the clustering effect. When combining the models with the BFI approach, we must take this clustering into account. New expressions for the BFI estimators have been derived (Appendix II.C). For *K* giving the number of clusters, the resulting BFI model has categorical specific intercepts: 

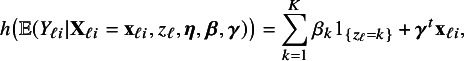

with 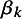 the intercept for the 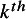 cluster, 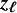 represents the cluster of center 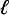 , and 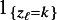 is an indicator function that equals 1 if 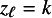 and 0 if 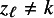 . As before, this model can be easily reformulated to a model with an intercept and a reference group.

### 3.6 Heterogeneity due to center-specific covariates

Covariates that are included in the local models are also included in the aggregated BFI model. If a variable does not vary within a center (e.g., the size of the medical staff or the percentage of female patients) it can not be included in the regression model for the center and is, therefore, not automatically included in the BFI model. The effect of such a variable is then hidden in the intercepts of the local models. In this subsection we explain how the BFI approach can be adjusted to estimate a (combined) BFI model that includes this center-specific covariate. Although the problem is the same for categorical and continuous variables, the statistical solutions are not. This has to do with the way the variable is included in the aggregated BFI model. If the variable is categorical, one or more binary dummy variables need to be included in the model to represent every category (minus 1). If the variable is included in the model as a continuous variable, only one variable needs to be included (under the assumption of linearity) that holds for all centers.

If the variable is categorical and every center has its own specific category, we are in the situation as described in Section 3.2, where the aggregated model has a center-specific intercept. If the number of categories is lower than the number of centers and multiple centers are in the same category, we actually have to deal with clustering as described in Section 3.5.

If the center-specific variable is continuous, for example the number of patients that is yearly treated in the corresponding center or the percentage of female patients, we actually want to fit a BFI model (based on all data) of the form: 
(6)

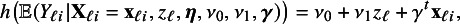

where 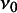 is the intercept, 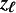 is the continuous center-specific variable, and 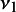 its corresponding unknown regression coefficient. The question is how to estimate the model parameters, and especially 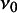 and 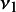 . This is explained below.

First all local models without this variable are fitted as described before. Next, the models are combined with the BFI methodology under the assumption that all intercepts may be different (the calculations are given in Appendix II.B and is also explained in Section 3.2). This yields an estimate of the model with a center-specific intercept: 

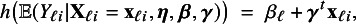

for center 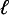 . The effect of the continuous variable is hidden in this intercept: 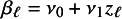 . To estimate 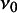 and 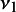 based on the estimated intercepts 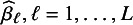 and 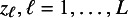 , one could make a scatter plot of the points 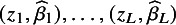 . Next, after fitting the least squares line through the points, the parameter 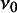 can be estimated by the intercept of the least square line and 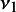 by its slope. This approach ignores differences in the precision of the estimates of the hospital-specific intercepts. This precision can be taken into account as follows. For sufficiently large samples, the (local) MAP estimators are approximately normally distributed, with a mean and a variance that can be estimated as described in the article. For each center, a value is randomly drawn from this distribution and based on the obtained values, 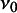 and 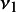 are estimated as described above. This procedure is repeated many times (*B*), yielding *B* estimates of 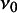 and 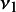 . Final estimates for 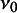 and 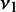 can be computed by taking their averages.

### 3.7 Asymptotic performance of the BFI estimator under heterogeneity

For both the homogeneous and the heterogeneous settings, the asymptotic distributions of the BFI estimators are derived in Appendix III. In the homogeneous setting, it turns out that the BFI estimator is asymptotically zero-mean Gaussian with covariance matrix equal to the inverse of the Fisher information matrix; the BFI estimator is asymptotically efficient. This distribution is equal to the asymptotic distribution of the MAP and maximum likelihood estimators that would have been based on the combined data; hence asymptotically no information is lost if the data cannot be merged.

In the heterogeneous setting with center-specific parameters, the parameters of interest can be split into those that are the same between the centers and that are center-specific. Expressions of the corresponding BFI estimators are given in (A.9) and (A.10) in Appendix II.B. In Appendix III.C it is proven that both BFI estimators are asymptotically Gaussian with covariance matrices that equal those for the MAP estimators and MLEs that would have been based on the combined data. Also in the heterogenous setting the BFI estimators are asymptotically efficient. Again asymptotically no information is lost if the data sets cannot be combined. In Appendix III.C it is proved that the BFI estimator for the center-specific parameter is asymptotically more accurate than the MAP estimator based on the local data of the center only. This is because the BFI estimator uses information from all centers to estimate the parameters that are the same across centers, while the MAP estimator uses local data only. A more accurate estimate of the shared parameters leads to a more accurate estimate of the non-shared parameters.

Expressions of the BFI estimators for the setting in which the centers can be clustered are given in Appendix II.C. These expressions are complicated. Therefore, the derivation of the asymptotic distribution is not given here, but can be derived in the same way as for the setting with center-specific parameters.

Since the BFI estimator of 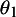 is asymptotically Gaussian and the asymptotic covariance matrix can be estimated by the inverse of 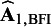 , credible intervals can be easily constructed, as explained for the homogeneous setting. Hypotheses can be tested using the Wald test.

### 3.8 Methods for checking heterogeneity

In this article we extend the BFI methodology to account for heterogeneity between centers. Before combining the local estimates, we should verify whether this heterogeneity is actually present and whether it is necessary to account for it.

Suppose we want to investigate whether it is necessary to take into account the heterogeneity of the intercepts. Then, first the MAP estimates of the local intercepts, say 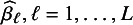 , should be compared. However, there will always be differences between the estimates. The question is whether the observed differences are due to randomness or whether the true values of the intercepts are sufficiently different to take this into account in the modelling. The latter can be verified by constructing credible intervals. In order to compare the parameter estimates between two centers, say centers *k* and 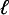 , a credible interval for the difference of the two intercepts can be constructed. Such a calculation is based on the statistical independence of the estimators 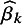 and 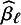 (since the data from the different centers are assumed to be independent) and the fact that 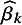 and 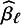 are approximately Gaussian with mean 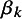 and 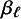 and standard deviations 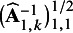 and 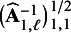 , respectively, (if the first element of the parameter vectors 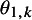 and 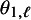 correspond to the intercept). Then, the 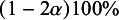 credible interval for the difference 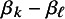 equals 

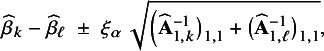

for 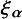 equal to the upper 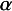 -quantile of the standard Gaussian distribution. With the latter interval we can verify whether the parameters in the centers *k* and 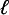 are different. If the sample sizes in the centers are small, the credible intervals may be wide and it may be difficult to conclude on hetereogeneity.

Similarly, the 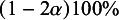 credible intervals for the difference between the true 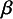 -value in all centers except 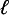 and the true parameter value in center 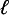 equals: 

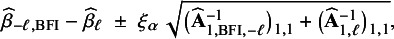

where subscript 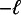 means that the BFI estimator was computed not including the estimator from center 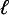 . With this interval we can verify whether the intercept in center 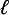 differs from the intercepts in the other centers assuming that these intercepts equal.

In the same way, one can check whether it is necessary to take into account any of the other types of heterogeneity.

## 4 Performance of BFI methodology

The BFI methodology for GLMs was introduced in Jonker et al^23^ and extended to survival models for homogeneous populations in Pazira et al.^25^ Simulation studies in those papers show good performance of the methodology in the homogeneous setting. In this article we focus on different types of heterogeneity. The results of simulation studies (Section 4.1) and data analyses (Section 4.2) are described below.

### 4.1 Simulation studies

#### 4.1.1 One-shot estimators for comparison

As explained in the introduction, we are only interested in one-shot estimators, i.e., estimators that can be calculated after a single communication with the centers, like the BFI estimator. To enable performance comparison for the BFI estimator, we consider two one-shot estimators. The most interesting one is the weighted average estimator (WAV) which is defined as the weighted average of the local MAP estimators with the weights equal to 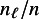 (where 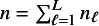 ); estimates based on larger data-sets are given larger weights. The weighted average estimator for 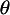 is defined as: 

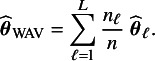

In case of clustering, the WAV estimator for the parameter that is specific for a particular cluster is defined as the weighted average of the local MAP estimators of the centers in that cluster. If a parameter may vary between all centers, the corresponding WAV estimator is defined as the MAP estimator in the local center. The second one-shot estimator for 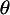 is the single center estimator 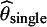 , defined as the MAP estimator in the center with the largest local sample size. The single center estimator cannot be defined in case of center or cluster specific parameters.

In Appendix III the asymptotic distributions of the weighted average and the single center estimators are derived. As expected, the asymptotic variance of the single center estimator is larger than the one of the BFI estimator, because it is based on fewer data points. In the homogeneous setting, the WAV estimator turns out to be asymptotically efficient (minimum variance) and it follows asymptotically the same distribution as the BFI estimator. In the heterogeneous setting, the WAV estimator of the parameter that differs between the centers is defined as the (corresponding) single center estimator. As explained in Section 3.7, the BFI estimator has a smaller asymptotic variance than this estimator. In this section the finite sample behaviour of the estimators are compared by means of simulation studies.

#### 4.1.2 Performance measures for finite samples

Since the BFI methodology tries to reconstruct from local inferences what would have been obtained if the data sets had been merged, the BFI estimators by definition cannot do better than the MAP estimators based on the combined data. Therefore, the parameter estimates and outcome predictions obtained by the BFI approach are compared to those found after combining the data. For completeness, we also compare the estimates with the true parameter values.

In the next subsection, the simulation procedure is explained. In brief, *B* times data sets are simulated from a chosen model, for every center separately. In every cycle the parameters are estimated with the three one-shot estimators, and also by the MAP estimator based on the combined data. Performance is measured with the 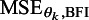 defined as 

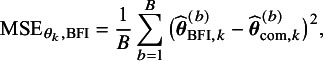

where 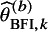 is the BFI estimated value of the *k*th coordinate of 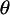 in the *b*th iteration, and 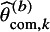 the estimate using all data. The MSE’s for the other estimators are defined similarly: 

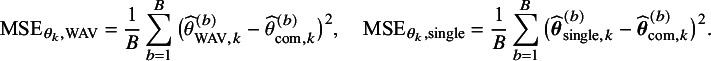

If the MSE is small, the estimates based on the local inference results are similar to the estimates based on the combined data, and thus only little information is lost. For the BFI estimator we also computed the MSE compared to the true parameter value; denoted as 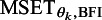 (where the *T* stands for “true value”).

#### 4.1.3 Simulation settings and results

We assume that there are four centers with data of 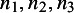 , and 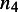 individuals. For each individual, data of three independent covariates are simulated: two from a Gaussian distribution and one from a binomial distribution. The outcome variables given the covariates are assumed to be independent and are simulated from a logistic regression model. We consider the following situations: 1) the populations are homogeneous, 2) the distributions of the covariates differ across the centers, 3) the intercepts (prevalence) differ across the centers, and 4) centers are clustered. For the sample sizes we consider two settings: small sample sizes ( 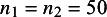 , 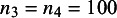 ) and large sample sizes ( 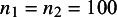 , 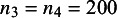 ) and we set the covariance matrices of the Gaussian prior equal to diagonal matrices with 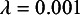 or 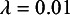 (or a mix) on the diagonal. This corresponds to variances that equal 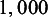 and 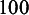 respectively; the prior distributions are almost non-informative. The first covariate is sampled from a Gaussian distribution with mean zero and standard deviation equal to 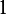 . The second covariate is Gaussian as well, but with mean 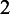 and standard deviation 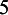 . The third covariate comes from a binomial distribution with probability 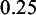 . In the setting with heterogeneous populations, different covariate distributions have been used across the centers. In all cases the regression parameters equal 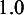 for the intercept and 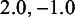 , and 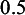 for the three covariates.

**Table 1:** Homogeneous setting

			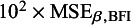					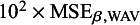					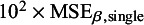					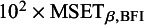			
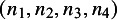	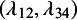		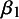	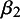	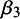	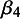		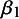	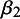	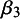	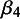		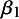	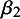	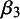	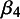		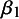	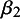	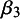	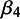
(25,25,50,50)	(0.001, 0.001)		14.42	41.14	9.81	24.52		496.6	1322	387.6	325.4		997.9	2495	636.5	1069		21.36	35.42	6.81	59.52
	(0.01, 0.01)		10.83	32.6	8.11	14.92		79.93	198.8	65.92	64.8		229.5	432.2	103.4	387.2		21.03	36.28	6.62	64.46
	(0.01, 0.001)		12.76	36.91	9.36	17.37		271.7	760.1	219.9	224.8		1234	2485	686.2	1136		20.60	34.99	6.66	62.83
(50,50,100,100)	(0.001, 0.001)		3.54	11.47	2.74	3.73		74.45	225.8	63.5	77.21		62.93	279.17	51.37	196.6		8.38	14.58	2.72	24.62
	(0.01, 0.01)		3.51	9.91	2.55	2.73		18.50	45.73	12.43	18.67		44.10	81.51	15.89	94.96		8.56	14.02	2.62	24.50
	(0.01, 0.001)		3.60	10.89	2.74	3.33		19.72	57.89	15.27	20.66		50.25	119.2	25.19	105.9		9.14	14.22	2.54	26.64
(100,100,200,200)	(0.001, 0.001)		0.77	2.49	0.61	0.54		3.25	10.49	2.25	2.87		10.80	16.10	3.05	31.73		4.39	6.32	1.11	11.72
	(0.01, 0.01)		0.80	2.64	0.64	0.60		2.21	6.94	1.64	2.76		10.92	19.25	3.08	34.88		4.21	6.30	1.07	11.92
	(0.01, 0.001)		0.82	2.72	0.66	0.60		2.12	6.60	1.52	2.22		12.78	21.11	3.87	34.10		4.43	6.25	1.08	13.02
(200,200,400,400)	(0.001, 0.001)		0.17	0.60	0.14	0.12		0.27	0.88	0.21	0.39		4.72	7.16	1.08	14.48		2.16	3.02	0.49	6.21
	(0.01, 0.01)		0.18	0.59	0.14	0.11		0.29	0.87	0.21	0.35		5.16	7.26	1.21	14.96		2.04	2.93	0.48	6.91
	(0.01, 0.001)		0.20	0.65	0.16	0.14		0.30	0.94	0.23	0.39		5.30	7.31	1.22	16.31		2.00	2.87	0.50	6.53

*Note:* The MSEs for the BFI, weighted average and the single center estimators, and MSET for the BFI estimator. The prior inverse covariance matrices are diagonal with the diagonal element equal to 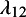 in centers 1 and 2, and the value 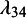 in centers 3 and 4. The single center estimates are based on data from center 4 only.

**Table 2:** Heterogeneous setting

			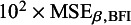					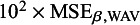					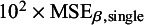					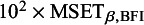			
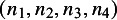	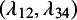		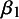	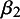	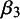	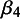		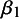	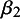	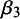	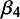		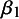	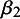	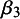	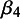		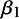	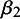	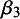	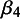
(50,50,100,100)	(0.001, 0.001)		3.22	8.96	2.28	3.27		133.8	169.9	38.34	100.3		1093	1245	272.2	879.9		14.33	9.93	2.76	19.63
	(0.01, 0.01)		3.31	8.55	2.27	2.89		64.24	46.38	10.43	35.90		534.5	242.9	53.92	340.5		14.71	9.35	2.75	20.03
	(0.01, 0.001)		3.01	8.20	2.09	2.65		107.7	128.2	27.23	73.06		955.5	923.5	192.5	668.4		14.62	9.84	2.59	18.75
(100,100,200,200)	(0.001, 0.001)		0.64	1.82	0.49	0.53		14.99	6.15	1.10	9.42		125.5	38.34	6.43	115.4		8.30	4.09	1.21	10.13
	(0.01, 0.01)		0.66	1.84	0.50	0.51		16.26	7.50	1.37	8.21		148.7	49.35	9.34	100.80		7.48	4.03	1.16	9.74
	(0.01, 0.001)		0.60	1.87	0.49	0.63		17.03	7.15	1.35	11.62		149.1	50.89	9.37	134.70		7.86	4.20	1.28	9.81

*Note:* The MSEs for the BFI, weighted average and the single center estimators, and MSET for the BFI estimator. The distributions of the covariates differ across the centers. The first covariate is Gaussian with mean 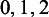 , and 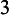 in the four centers, and standard deviation 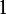 . The second covariate is Gaussian as well with mean 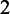 , but now the standard deviation varies: 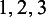 , and 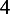 in the four centers. The third covariate comes from a binomial distribution with probability 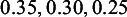 , and 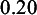 in the four centers. In all cases the prior inverse covariance matrix equals diagonal matrices with the diagonal element equal to 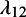 in the centers 1 and 2, and the value 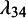 in the centers 3 and 4. The single center estimates are based on data from center 4 only.

For every setting, we simulate 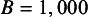 data sets, compute the BFI, weighted average and single center estimates (the latter one only if relevant), and compute the MSEs. The simulation results in the four different settings are given in Table 1 (homogeneity between centers), Table 2 (different covariate distributions), Table 3 (different intercepts between centers) and Table 4 (clustering).

**Table 3:** Heterogeneous setting

	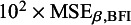								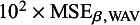								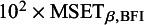						
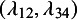	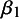	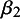	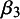	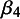	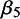	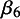	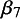		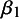	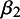	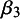	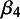	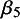	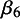	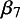		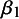	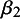	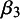	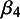	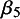	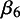	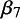
(0.001, 0.001)	7.45	10.29	2.41	4.21	14.33	3.60	5.90		703.17	1193.88	24.94	163.43	352.73	94.05	103.72		42.35	44.94	19.27	22.94	12.47	3.04	32.63
(0.01, 0.01)	4.00	6.66	1.96	3.39	12.41	3.08	4.10		120.80	266.14	21.43	46.34	73.79	19.59	25.15		41.36	42.99	20.09	20.96	11.7	2.82	34.23
(0.01, 0.001)	4.77	7.76	2.49	4.63	14.04	3.48	5.29		130.48	241.2	51.51	230.56	186.45	50.05	71.33		36.99	40.48	19.13	23.19	11.47	2.71	33.52
(0.001, 0.001)	0.72	1.36	0.44	0.76	3.19	0.80	0.82		37.03	80.79	4.82	8.34	19.85	5.08	7.32		18.80	19.56	9.64	9.92	5.24	1.27	15.63
(0.01, 0.01)	0.44	1.20	0.36	0.75	3.02	0.75	0.96		19.57	46.91	4.93	7.26	8.88	2.26	4.27		17.85	19.10	9.79	10.87	5.49	1.30	16.05
(0.01, 0.001)	0.45	1.30	0.41	0.79	3.14	0.78	0.91		25.72	47.00	5.71	8.38	11.27	2.81	4.72		17.35	19.37	9.51	11.59	5.26	1.19	15.77

*Note:* The MSEs for the BFI and weighted average estimators, and MSET for the BFI estimator. The intercepts differ across the centers. The parameters 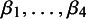 are the center-specific intercepts for the four centers (with true values 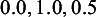 , and 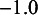 ). The parameters 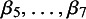 are the regression coefficients for the three covariates. For the upper three lines in the table, the local sample sizes equal 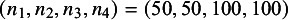 , and for the lower three lines they equal 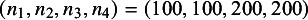 . The MSE for the single center estimator has been left out, because this estimator can estimate one intercept only.

**Table 4:** Heterogeneous setting

			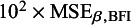						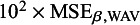						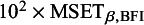				
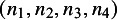	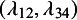		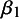	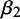	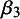	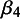	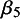		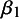	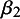	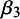	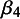	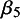		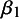	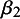	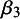	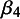	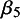
(50,50,100,100)	(0.001, 0.001)		8.17	14.82	13.69	3.38	5.45		814.44	151.48	333.89	86.33	114.08		24.23	20.82	11.71	2.97	30.59
	(0.01, 0.01)		5.24	12.91	12.21	3.03	4.24		126.42	44.30	62.15	16.21	26.07		23.92	21.60	12.55	2.94	32.55
	(0.01, 0.001)		5.55	13.08	12.35	3.14	4.35		136.91	105.72	92.36	24.71	31.79		23.71	22.37	12.45	3.06	34.42
(100,100,200,200)	(0.001, 0.001)		1.22	3.34	3.19	0.78	0.90		44.17	6.49	15.55	3.79	7.41		9.59	9.54	4.89	1.11	14.02
	(0.01, 0.01)		1.19	3.07	2.97	0.73	0.72		24.54	6.49	8.54	2.12	3.79		10.06	9.56	5.16	1.25	14.4
	(0.01, 0.001)		1.30	3.38	3.21	0.79	0.75		26.25	7.23	9.38	2.24	3.65		10.54	10.28	5.45	1.31	15.02

*Note:* The MSEs for the BFI and weighted average estimators, and MSET for the BFI estimator. The centers 1 and 2 and the centers 3 and 4 are clustered. The parameter 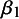 and 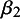 are the cluster specific intercepts (true values 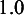 and 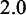 ). The parameters 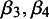 and 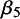 are the regression parameters of the three covariates. The MSE for the single center estimator has been left out, because the intercept for a single cluster can be estimated.

From the results in the tables it can be seen that for all estimators the MSEs decrease for increasing sample size. For the BFI estimator the decrease is stronger for the MSEs than for the MSETs. A decrease is as expected as a larger sample size yields more accurate estimates.

Further, the results show that the MSEs for the BFI estimates are smaller than those for the weighted average and the single-center estimates. This also holds MSET (the 
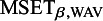
 and 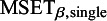 are left out from the tables, due to a lack of space.) The relative differences between 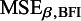 and 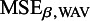 decrease with increasing sample size. This was expected, as (in the homogeneous setting) the asymptotic distributions of the BFI and the WAV estimators are identical. For finite sample sizes the differences in MSE are still considerable.

In all settings the 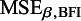 is smaller than the 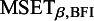 . This is as expected, as the randomness in the observations is reflected in the estimate based on the combined data, but not in the actual parameter values. An important observation is that within every setting and for all combinations of sample sizes, the 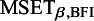 is rather stable for the different combinations of 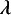 -values. Since the actual parameter values are independent of the choice of 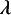 , we can conclude that the BFI estimates are not very sensitive to the values of 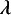 (considered here). However, the MSE for the three estimators decreases (slightly) for increasing 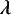 (a smaller prior variance), especially when the sample size is small. For larger values of 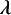 , the MAP estimates have shrunk further to zero, leading to smaller MSEs. The latter does not imply that the estimates are more similar to the actual values.

When comparing the MSEs of the different regression parameters (within the same setting and set of sample sizes), it is clear that some regression parameters can be estimated more accurately than others. For example, comparing the MSEs for the regression coefficients of the first and the second covariate (i.e., for 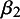 and 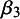 in Table 2) it can be seen that the MSEs for the coefficients for the second covariate are smaller, probably because the variation in this covariate is larger than in the first one. This applies to all estimators. When comparing the values of 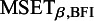 in Tables 1 and 2, we see that the estimates of the regression parameters (except the intercept) are more accurate in the heterogeneous setting with different covariate distributions across the centers. Again, this is probably due to the increased variation in the covariate values. An opposite effect is seen for the WAV estimator; more variability in the covariates leads to larger MSE.

Also in settings with center-specific intercepts (different prevalence in the centers) and clustering, the BFI estimators clearly perform better than the weighted average estimators, this is especially true for the center-specific and the cluster specific intercepts. Within the BFI methodology, these estimates use information from all centers for estimation. This is not the case for the weighted average estimator.

### 4.2 Data analysis

#### 4.2.1 Description of the data

The data come from a hypothetical study on stress among nurses in hospitals.^26^ The data set consists of simulated data of 1,000 nurses working on different wards in 25 hospitals.^iii^ The outcome of interest is job-related stress among nurses. Additionally, for every nurse the following variables are available: age (years), experience (years), gender (0 = male, 1 = female), the type of ward in which the nurse works (0 = general care, 1 = special care), hospital (1, 2, …, 25), and hospital size (small, medium, large). In the data, the number of nurses per hospital runs from 36 to 52. Further, for the covariates, the averages of the ages of the nurses in the different hospitals run from 39.2 to 46.3 years, the fraction of female nurses from 0.61 to 0.85, the number of years of experience from 14.9 to 18.5, and the fraction of nurses on a special care ward runs from 0.48 to 0.51. For some of these variables there is hardly any variation across the hospitals, whereas for other variables the variation is much larger, like the fraction of female nurses. So, there seems to be some heterogeneity of the population characteristics across the centers (see Section 3.1). Further, there are nine small hospitals, 12 medium sized hospitals, and four large hospitals. The stress level in hospitals seems to increase with the size of the hospital; there is heterogeneity due to a hospital size clustering effect (see Section 3.5). The variation of the stress levels of nurses in the data across the centers (the averages vary between 3.6 and 5.8) may also be due to non-measured hospital effects like location and patient population (see Sections 3.2 and 3.6). In every hospital we fitted a linear regression model and estimated the variance of the error term. The estimated variances vary from 0.17 to 1.16. It seems that there may be heterogeneity in this variance parameter (see Section 3.4). In Section 4.2 we estimate linear regression models with the BFI methodology, adjusted for these types of heterogeneity.

For better comparison and interpretation of the estimates of the regression parameters, we standardized the continuous variables age, experience and stress: from each observed value we subtracted the full sample mean and divided the result by its full sample standard deviation. This is not required for the BFI method. However, note that this can be easily done without combining all data, since the full sample mean and standard deviation can be easily reconstructed from the local sample means and local standard deviations (and thus only these values need to be shared with the central server).

#### 4.2.2 Model estimation under heterogeneity

In this subsection we analyse the data from the 25 centers with the BFI methodology and we compare the estimated aggregated BFI model to the model that would have been found if the data had been combined before fitting the model. As described in the previous subsection we have different types of heterogeneity. We start with a relatively simple linear regression model and combine the local MAP estimates with the BFI methodology under the assumption of homogeneity across the centers. In a second analysis we also include a clustering effect for the variable hospital size, in the third analysis we allow a center-specific intercept, and in the last step we also allow for difference variances of the error term. In Appendix I it is explained how these analyses can be performed in R with our R-package BFI.

In the first analysis we only include nurse-specific variables: age, gender, experience (exp), and wardtype. We fit a linear regression model of the form: 

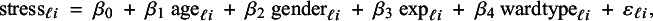

where the subscript “ 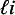 ” refers to the 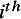 person in center 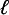 . The last term, 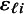 , is the measurement error in the outcome variable, which is assumed to be Gaussian with mean zero and variance 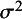 . In the analyses based on the combined data and in the centers we take Gaussian priors with a diagonal inverse covariance matrix 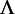 with either 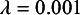 or 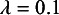 on the diagonal. For these values of 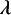 the corresponding variances of the parameter priors are equal to 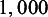 and 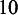 , respectively. For a prior variance equal to 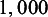 , the MAP estimates are close to the maximum likelihood estimates, since the prior density is almost flat.

**Table 5:** The BFI estimates of the parameters in the linear regression model, 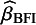 , and the MAP estimates obtained from the analysis after combining the data, 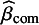

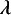		intercept	age	gender	experience	wardtype	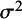
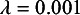	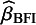 (sd)	0.522 (0.043)	0.263 (0.034)	−0.502 (0.044)	−0.386 (0.034)	−0.011 (0.039)	0.539
	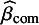 (sd)	0.332 (0.066)	0.233 (0.052)	−0.503 (0.068)	−0.352 (0.052)	0.075 (0.060)	0.907
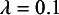	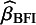 (sd)	0.523 (0.043)	0.264 (0.034)	−0.503 (0.044)	−0.386 (0.034)	−0.011 (0.039)	0.537
	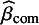 (sd)	0.332 (0.066)	0.233 (0.052)	−0.503 (0.068)	−0.352 (0.052)	0.075 (0.060)	0.907

*Note:* The corresponding estimated standard deviations (sd) are given within the brackets. The prior inverse covariance matrices are diagonal with the diagonal elements equal to either 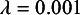 or 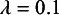 . In the last column the estimates of 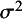 , the variance of the error term, are given.

The results are given in Table 5. It can be seen that the value of 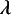 hardly effects the estimates of the parameters; possibly because the total sample size is high. The BFI estimates for the regression parameters for the covariates age, gender and experience are similar to those obtained based on the combined data. For wardtype the estimates are close in absolute sense, but from the estimates and the relative large standard deviations it is clear that the contribution of this covariate to the model is minimal. The estimates of the intercept and the variance of the error term, 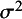 , seem to differ substantially. This is possibly caused by the presence of heterogeneity across centers (e.g., varying hospital size and variances) for which is not corrected in the models (but will be in the next analysis). In the centers, the hospital size is taken into account via the intercept. This leads to different estimates of these intercepts across the centers and small variances of the error term. The BFI methodology combines the local estimates to a single estimate under the incorrect assumption of homogeneity, which leads to the differences of 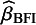 and 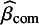 . In the next analysis, heterogeneity due to varying hospital sizes is taken into account and we will see that the differences between the estimates obtained with the two procedures will (almost) disappear. For the BFI methodology, but also if pooled data is available, it is important to correct for possible heterogeneity. We moreover leave out the covariate wardtype from the model.

Because the size of the hospital is predictive for the stress level, we want to add this variable to the model as well. This variable is a categorical variable with three categories (small, medium, large). For the combined data, the linear regression model that includes the variable hospital size via category specific intercepts, is given by: 

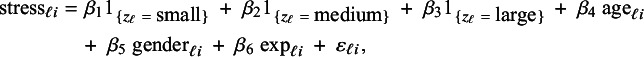

with 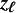 the category of the hospital size in hospital 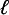 (so small, medium or large) and 
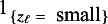
 is defined as 1 if hospital 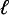 is small and zero otherwise. The functions 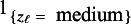 and 
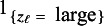
 are defined in a similar way. There is no general intercept in the model; this is hidden in the three intercepts. The model can be reformulated in a model that includes a general intercept (as was explained in Section 3). To obtain a BFI aggregated model with category specific intercepts, we apply the BFI approach as described in Section 3.5. The estimates are given in Table 6. From the results we see that the estimates of the regression parameters obtained with the BFI methodology are very similar to those obtained based on the combined data; also for the three intercepts 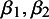 , and 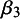 . However, there are still some differences between the estimates for 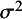 , but these are smaller than in the first analysis. Possibly more (unknown) variables need to be included in the model or there is heterogeneity in the variances across the centers. From the estimates of the intercepts, it is clear that there is a positive relationship between stress and the size of the hospital (adjusted for the other variables in the model): nurses in large hospitals seem to experience more stress than nurses in small hospitals.

In a third analysis we include a hospital specific intercept in the model. Now, the variable hospital size is redundant as this effect is included in the hospital effect. The model for the combined data is given by: 

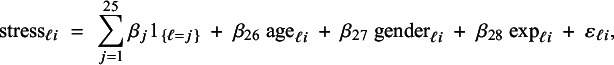

with 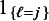 an indicator function defined as 1 if hospital 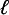 is the 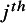 hospital and zero otherwise. That means that for hospital 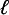 , 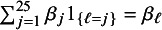 . So, every hospital has its own specific intercept and there is no general intercept. We fit the model after merging the data and by combining the estimates in the different hospitals with the BFI methodology, as described in Section 3.2. The results are given in Table 7. Since the number of intercepts is large (for each hospital one intercept), we decided to leave out these estimates from the table, but made a scatter plot instead for comparison (not presented). The plot shows almost perfect agreement between the estimated intercepts based on the BFI methodology and the estimates found after combining the data. The estimates of the remaining parameters obtained with the two estimation procedures, shown in Table 7, show nice agreement as well; also for the variance 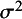 .

**Table 6:** The BFI estimates of the parameters in the linear regression model with a cluster effect for hospital size, 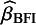 , and the MAP estimates obtained from the analysis after combining the data, 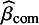

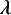		I(small)	I(medium)	I(large)	age	gender	experience	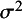
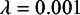	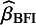 (sd)	0.004 (0.058)	0.497 (0.043)	0.958 (0.061)	0.270 (0.035)	−0.478 (0.046)	−0.381 (0.036)	0.581
	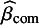 (sd)	−0.024 (0.066)	0.490 (0.063)	0.917 (0.086)	0.237 (0.049)	−0.493 (0.064)	−0.352 (0.049)	0.799
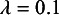	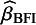 (sd)	0.004 (0.057)	0.497 (0.043)	0.958 (0.061)	0.270 (0.035)	−0.478 (0.046)	−0.381 (0.035)	0.580
	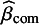 (sd)	−0.024 (0.066)	0.490 (0.063)	0.916 (0.086)	0.237 (0.049)	−0.492 (0.064)	−0.352 (0.049)	0.799

*Note:* The estimated standard deviations (sd) are given within the brackets. The prior inverse covariance matrices are diagonal with the diagonal elements equal to either 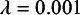 or 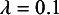 . The abbreviations “I(small)”, “I(medium)” and “I(large)” stand for the three intercepts for the categories small, medium, large. In the last column the estimates of 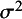 , the variance of the error term, are given.

Next, we consider the situation with heterogeneity in the variance of the error term in the linear regression model. We allow center-specific intercepts and center-specific variances of the error term in the model. The estimates of the regression parameters hardly change (data not presented here). Taking into account heterogeneity across the centers can improve the results, but increases the number of model parameters that need to be estimated.

#### 4.2.3 Prediction

In the previous subsection we studied the performance of the BFI methodology for estimating the model parameters. In this subsection we focus on prediction.

##### 4.2.3.1 Heterogenous populations

To study the performance of a prediction model that has been estimated with the BFI strategy, we follow the steps:

1. In every hospital we randomly select the data of approximately 10% of the nurses for the test-set. The remaining data form the training-set. The data of the nurses in this set will be used to estimate the BFI prediction model. The data in the test-set will be used to test the performance of the model.
2. In every hospital we compute the MAP estimates of the model parameters based on the local data from the training sets only.
3. Based on the inference results from the hospitals, we compute the BFI estimates of the model parameters with the BFI methodology.
4. Based on this estimated BFI model we predict the outcome (stress level) of the nurses in the test sets based on their covariate values. The prediction for the *i*th nurse in the 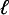 th hospital is denoted as 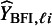 .
5. Parallel to this, we merge all data from the training sets and fit the regression model by MAP estimation.
6. With this model we predict the outcomes of the nurses in the combined test data set based on their covariate values. The predicted outcome for the *i*th nurse from hospital 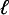 is denoted as 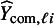 .
7. We plot the points 
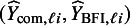
 in a scatter plot.

**Figure 1:**
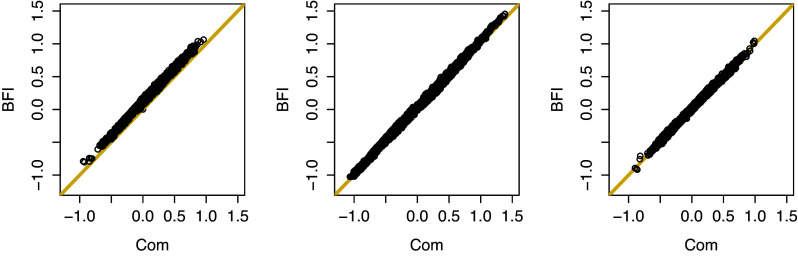
Outcome predictions based on the BFI strategy (vertical axis) versus those based on the MAP estimates from the analysis obtained after combining the training data sets (horizontal axis). Left: Heterogeneous populations. Predictions are based on the model that includes the covariates age, gender, experience. Middle: Heterogeneous populations. Predictions are based on the model that includes the covariates hospital size, age, gender, experience. Right: Homogeneous populations. Predictions are based on the model that includes the covariates age, gender, experience. Perfect agreement corresponds to all points on the diagonal (yellow line). Here, 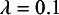 . The plots look similar for other values of 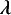 .

The steps above are repeated 50 times and all points are plotted in the same figure, see Figure 1 for three different settings. The predictions in the plot on the left were found based on the fitted model with the covariates age, gender, and experience. For the plot in the middle the covariate hospital size was included as well (as described in Section 3.5). In both cases 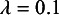 . From the left plot we see that for the model that does not include the covariate hospital size, the BFI predictions are slightly higher than those found with the prediction model estimated based on the combined data. This is caused by the estimates of the intercept; in Table 5 we already had seen that the intercept in the model fitted with the BFI method is higher than the estimated intercept in the model based on all data. This difference is due to heterogeneity of the data that is not taken into account in the model (see Section 4.2 for a discussion). After adding the variable hospital size to the model this discrepancy disappears and there is a very strong agreement between the predictions obtained with the two methods. The variation in the predictions has increased which indicates a higher explained variance by the inclusion of the variable hospital size.

**Table 7:** The BFI estimates of the parameters in the linear regression model with hospital specific intercepts, 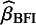 , and the MAP estimates obtained from the analysis after combining the data, 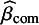

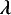		age	gender	experience	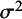
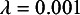	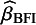 (sd)	0.268 (0.036)	−0.452 (0.047)	−0.364 (0.036)	0.581
	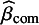 (sd)	0.247 (0.043)	−0.474 (0.057)	−0.357 (0.044)	0.614
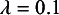	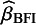 (sd)	0.268 (0.036)	−0.452 (0.047)	−0.364 (0.036)	0.580
	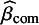 (sd)	0.247 (0.043)	−0.471 (0.057)	−0.357 (0.044)	0.614

*Note:* The corresponding estimated standard deviations (sd) are given within the brackets. The 25 estimated intercepts are not presented in the table. The prior inverse covariance matrices are diagonal with the diagonal elements equal to either 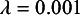 or 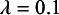 . In the last column the estimates of 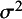 , the variance of the error term, are given.

##### 4.2.3.2 Homogeneous populations

In the previous subsection we considered prediction accuracy of the BFI prediction model based on the data of nurses from the 25 hospitals. As mentioned before the nurses in the different hospitals may come from different populations. In this subsection we aim to study the performance of the BFI prediction model for homogeneous (nurse) populations. To be sure that the populations are homogeneous we randomize all nurses over the hospitals, keeping the sample sizes in the hospitals fixed. Now, the populations in the different hospitals can be seen as samples from the same population. Next, we follow the steps given in the previous subsection. This, including the randomization, is repeated 50 times. The variables we included in the model are age, gender, and experience. It can be seen that the agreement between the predictions is very strong. The scatter plot on the left in Figure 1 was obtained for the same model, but for the heterogeneous setting. In that case we saw some discrepancy between the predictions from the two models. Since this is not seen in the homogeneous setting and also not in the scatter plot for the models that take the hospital size into account, we conclude that the discrepancy was due to the heterogeneity that was not taken into account in the first simulation.

## 5 Discussion

In this article, we have extended the BFI methodology for homogeneous to heterogeneous populations. The aim of the BFI methodology is to construct from the inference results obtained in multiple separate centers, what would have been found if the analysis had been performed on the combined data set. The key merit is that no individual data need to be transferred from the local centers to a central server. As a consequence, Data Transfer Agreement (DTA) for data sharing, can be simplified significantly. This may improve collaboration between researchers from different institutes and accelerate research.

In the BFI framework, statistical models are fitted in the separate centers based on local data only. So, in every center someone with sufficient knowledge of statistics and R needs to be available to do the analysis. Of course, the statistician who is concerned with combining the separate inference results can assist and can even provide code to be sure that the analyses in the separate centers are consistent. It is therefore important that a single communication with the local centers is sufficient to calculate the BFI estimators.

For different types of heterogeneity, new expressions of the BFI estimators have been derived. Asymptotically, the BFI estimators have been proven to be efficient (minimum variance) and we show that no information is lost as a consequence of the fact that the data cannot be combined. Simulation studies have shown that the performance of the BFI estimator is also good for finite samples, and better than that of the weighted average estimator. Furthermore, in this article it is explained how to do the analyses in R with the software package BFI that we developed to make the methodology easily accessible for the user. The mathematical details are given in two appendices, and can be ignored if one’s interest is solely in the application of BFI.

It may happen that communication between the central server and some data centers is intermittent or delayed. In that case, the BFI estimators can be calculated based on the estimation results available so far. As soon as more centers have sent their results, the BFI estimators can be recalculated, including the results from the delayed centers. This can be easily done by using the expression of the BFI estimators and will result in exactly the same final estimate compared to the estimate that would have been found if all centers had sent their local estimates at the same time. Also, if a center wishes to participate in the study at a later date, the BFI estimate can be easily updated, as just described. Ideally, however, it would be decided in advance which centers will participate in the study, to avoid researchers selecting centers based on local estimates. In many other federated analysis methods, in contrast, estimates are found by cycling around the centers and updating parameter estimates based on the local data. Then, if one or more centers are included in the estimation process at a later moment, the entire optimization process needs to be repeated, which can be a rather time-consuming process.

The prior of the parameters is taken equal to a zero-mean Gaussian distribution. This assumption allows the derivation of explicit expressions for the BFI estimators. For other prior distributions this may not be the case. If a Gaussian prior is not suitable for a parameter, for example because it is positive by definition, it can be transformed (e.g., via a log transformation). For example, for the variance of the error term in a linear regression model, the Gaussian prior for the log transformation of the parameter is used and implemented in the R package. The Gaussian prior corresponds to a ridge penalty, which is often used in practice to reduce overfitting. If one also wants to do selection, a lasso penalty is more common and a different prior distribution must be assumed. Then the BFI estimates must be found by numerical optimization.

For the centers different covariance matrices for the Gaussian prior may be chosen. One reason to do this could be the local sample size. The smaller the variance of the Gaussian prior, the more the estimates are shrunken to zero. Also if there is a difference in reliability of the data across the centers (data in some centers are “cleaner” than in others), different prior covariances can be used. It is up to the user to decide whether to assume equal priors or not.

The sets of variables available for fitting a regression model may differ across the centers. This happens, for instance, if some patients’ or individuals’ characteristics are measured and documented in most centers, but not in all. If a missing variable may be predictive for the outcome, a single or multiple regression method can be applied to impute the missing values.^27^ Then, a regression model with this missing variable as an outcome and the original outcome variable and the remaining variables as covariates is fitted, by applying the BFI approach in the centers in which this “missing variable” has been measured. Next, this estimated regression model is used to predict the variable values in the center in which the variable was not measured. After a single or a multiple imputation, the BFI strategy as described before can be used.

The BFI estimators are defined as the maximizers of an approximation of the log posterior density (second order Taylor expansions) for the merged data set. In the homogeneous case, these approximations are known to be accurate if the total sample size is sufficiently large (compared to the dimension of parameter space). However, if parameters are assumed to be distinct across centers, the local sample sizes need to be sufficiently large as well. If the total or local sample sizes are small or if the dimension of the parameter space is large, a higher order approximation (third or higher order of the Taylor expansion) may yield more accurate results. This and regularization methods to overcome overfitting will be studied in a new project. The same holds for the BFI estimates of the asymptotic covariance matrix and, thus, for the standard deviations.

The theory for the BFI approach has been developed for parametric models, including generalized linear models (GLMs) and survival models,^25^ and has been tested for multiple data sets. In case of possible (unmeasured) heterogeneity between centers, a multilevel model including random center effects and random slopes could be considered if the data from the different centers can be combined. The BFI methodology also applies to these types of models, but the corresponding BFI software has not yet been developed. Heterogeneity can be taken into account, as described in this article. The R package BFI will be continuously developed and will include multilevel models in the near future.^24^

The BFI methodology makes it possible to obtain the statistical power of the combined data set *without actually combining the data*. DTA’s can hence be simplified and collaboration between centers may increase.

## Data Availability

The data are available in the R package BFI. The R package BFI and a detailed manual are available on CRAN: https://CRAN.R-project.org/package=BFI. More information can also be found on the webpage: https://hassanpazira.github.io/BFI/.

## Notes

i. The theory in this article holds for any parametric regression model, but for simplicity of notation we focus on GLMs only.

ii. we use the letter *p* for any density. From the arguments it is clear which density is actually meant.

iii. The data are available in the software package BFI in R.

## Appendix I Bayesian Federated Inference in R

We have written the software package BFI in R for doing the BFI calculations.^24^ Here we explain how to do BFI analyses.

### MAP estimation

To compute the MAP estimates of the parameters in a regression model, the command MAP.estimation can be used. To apply this command, the data has to be in a specific form and the inverse covariance matrix of the Gaussian prior needs to be chosen. The analysis below is for the combined data set Nurse. The estimates in the separate hospitals can be obtained with the same commands, but with the local data sets instead.

The command inv.prior.cov creates a diagonal inverse covariance matrix for the prior distribution of the correct dimension. Based on the characteristics of the covariates (continuous or categorical) in M and the number of nuisance parameters, the number of model parameters is computed (the number of regression parameters for a categorical variable equals the number of levels minus one and a linear model (family="gaussian") has one nuisance parameter, the variance of the measurement error). The argument lambda=0.01 means that all elements on the diagonal of  equal 0.01. The arguments of MAP.estimation are the outcome variable Nurses$stress, the covariate data in M, the type of the model (family="gaussian") and the matrix Lambda. The inference results are stored in the list fit. A summary of it can be found with summary(fit).

When applying the BFI approach, the analyses are performed in every hospital and the results in fit are sent to the central server. There, the results from the different hospitals are combined. This is explained below.

### BFI for homogeneous populations

Suppose that all hospitals have sent their output to the central server. For ease of notation, we assume these outputs are stored in fit1, fit2, …, fit25. From every output the relevant elements need to be selected and combined. If the number of hospitals is high, it is easier to work with a for-loop. With the following code, all relevant elements of 25 local centers are created and combined by the main function bfi():

Here Lambda is the inverse covariance matrix of the prior for the (fictive) combined data. The command bfi combines the estimates from the different hospitals into the BFI estimates. The outcome fitbfi_homo is a list with the BFI estimates  and  . The command summary(fitbfi_homo) gives the BFI estimates (and more information).

### BFI for heterogeneous populations

Different types of heterogeneity have been discussed in Section 3. Below we will explain how to do the analyses in R.

Heterogeneity of population characteristics

Heterogeneity across population characteristics in the centers implies that the value of the parameter  differs across centers. Because the bfi-command estimates the parameter  (and its curvature matrix  ), and these estimates are not affected by  , the R-code explained in the previous subsection can still be applied.

Heterogeneity across outcome means

Suppose the intercepts differ across hospitals. To take this variation into account we allow a hospital specific intercept in the regression model. Instead of one general intercept there are  intercepts; an increase of  parameters. The dimension of the inverse covariance matrix Lambda for the fictive combined data set changes as well. For a diagonal matrix with 0.01 at the diagonal, this matrix can be obtained by

These commands replace the two corresponding commands above. The argument L=25 has to be added to indicate the number of centers, and thus the number of location specific intercepts. This matrix should be appended to the list priors instead. The MAP estimates can be obtained with the command bfi, but it needs to be made explicit that the hospitals may have different intercepts:

For this stratified analysis extra arguments have been added: stratified=TRUE and strat_par=1. The first argument indicates that the full model stratifies with respect to the different hospitals. The default is stratified=FALSE. If strat_par=1 there is stratification with respect to the intercept and if strat_par=2 this is the case for the variance of the measurement error in a linear regression model. A summary of the results can be obtained by |summary(fitbfi_hetero)|.This gives a list with estimates, starting with the hospital specific intercepts.

Heterogeneity due to clustering

An example of a cluster variable is hospital size. For all nurses in a hospital this covariate is constant and, as a consequence, the effect of hospital size on stress cannot be estimated within a hospital. However, the model for the (fictive) combined data could include this covariate if there is variation across the hospitals (Section 3.5). Here we explain how to do the analyses in R. In practice, every local hospital sends its size (small, medium, large) to the central server. Then, a vector with all sizes is defined in R. Suppose this vector is named Hsize. After fitting the local models (like explained before), the estimated model for the (fictive) combined data can be obtained with:

The commands return a list with categorical specific intercepts and the estimates of the remaining parameters.

## Appendix II Mathematical derivations of the BFI estimators

In this appendix the mathematical derivations of the BFI estimators are given for three settings:

- Appendix II.A: Homogeneity across centers.
- Appendix II.B: Heterogeneity across centers, center-specific parameter, e.g., the intercept.
- Appendix II.C: Heterogeneity across centers, due to clustering, e.g., geospatial regions.

### Appendix II.A Homogeneity across centers

In this appendix we derive expressions of the BFI estimators under the assumption that the variables  are independent and identically distributed. In equations (1) and (2) in Section 2 we have seen that the log posterior densities for the (fictive) combined data set  and for the subset  equal

(A.1)

(A.2)

By reordering the terms in equation (A.2), it follows that for every center

Next, summing over all centers yields

(A.3)

By inserting the right hand side of equation (A.3) into the right hand side of equation (A.1) this yields

(A.4)

We expressed the log posterior densities of the combined data,  , in terms of the log posterior densities of the local data sets,  . However, the final aim is to express the MAP estimator  based on the (fictive) combined data set  in terms of the MAP estimators based on the local data sets  . This will be done next. We approximate the log posterior densities for the data set  by a Taylor expansion up to the quadratic order in  around the MAP estimator  :

with  equal to minus the second derivative of  with respect to  , evaluated at  . The linear term in the Taylor expansion is equal to zero and therefore missing in the expansion; the MAP estimator maximizes the log posterior density and the first derivative evaluated at  is therefore equal to zero. The last term in the Taylor expansion is equal to  , where  represents a term that is bounded in probability for the sample size going to infinity.^28^ For  in a small neighborhood of  , the term  will be close to zero (in probability), and the remainder term  is small compared to the other terms in the Taylor expansion which are of an order of at most  .

Since the log posterior density in equation (A.2) is decomposed in terms that depend on either  or  , but never on both, the matrices  are diagonal block matrices:

with the blocks  and  equal to minus the second derivative matrices for  and  , respectively, and the log posterior densities can be approximated by

By substituting this expansion for  , into the relation (A.4), we obtain:

(A.5)

where *B* is a term that depends on the data, but is not a function of  . Now choose the prior densities  and  in the combined data set and  and  in center  to be Gaussian with mean zero and inverse covariance matrices  and  in the combined data set, and  and  in center  : e.g.,  . Inserting the expressions of the densities into (A.5) yields

(A.6)

for  a term that depends on the data, but not of  and  . The function  in equation (A.6) is quadratic function of  and  . Maximizing  with respect to  yields the BFI estimators

where  and  equal minus the second derivative of  with respect to  and  . In Appendix III.B the asymptotic distribution of the BFI estimators are derived.

### Appendix II.B Heterogeneity across centers, center-specific parameter

Suppose that the vector of regression parameters can be subdivided into two parts. One part is equal across the centers and the other part may vary. A special case is the situation in which the intercepts vary. In the calculations of the BFI estimator, we assume that the covariates are statistically independent between the individuals within and across the centers. We, moreover, assume that the outcome variables given the covariates and the center are independent.

Suppose that the vector  can be decomposed as  , where, as before,  is the vector of parameters that specifies the distribution of the covariates. The parameter  is decomposed so that  is the vector of (regression) parameters that is assumed to be equal across the centers, and  is the vector of (regression) parameters that may vary. In this appendix we consider the situation in which every center has its own specific vector of parameters:  for the *L* centers. The vector of parameters in the combined data set is equal to  , where  is the parameter vector in center  . If only the intercepts vary across the centers,  is one-dimensional, but for now we allow  to be a vector.

For simplicity of notation we assume that  and  are independent:  for the combined data set, and in center  :  . As before, the log posterior densities can be written as

and

Previously, and in the formulas above, we see that the log posterior density is decomposed into terms that depend on  or  , but never on both. That means that the BFI estimator for  is not affected by the estimator  and vice versa. Therefore, in this setting, the BFI estimator  can be expressed in terms of the local MAP estimators  and  as in (4). In the remainder of the derivation we focus on  only and leave out the terms with  from the expressions.

Like in the homogeneous setting, the log posterior density in the full data set can be written in terms of the local log posterior densities:

(A.7)

with *B* a term that depends on the data and on  , but is not a function of  .

Let  and  be the MAP estimators of  and  based on the data set  . Moreover, let  and  be minus the second derivative of  with respect to  and  respectively, and let  be minus the second derivative with respect to both  and  all evaluated at  .

The Taylor expansions up to the quadratic term of  around  is given by

Next, we insert the Taylor expressions into (A.7). For the combined data  we assume a Gaussian prior with mean zero and inverse covariance matrix  for  , and a zero mean Gaussian prior with inverse covariance matrix  for  . For center  , also zero mean Gaussian priors are chosen, but with inverse covariance matrices  and  . The dimension of  and  depends on the number of parameters that may vary across the centers. If only the intercepts vary, the matrices are scalars. After inserting these densities in expression (A.7) as well, we obtain

(A.8)

with  representing a term that does not depend on  . The function  is a quadratic function of  . Maximization of this function with respect to  by setting its derivative equal to zero, yields the BFI estimators

(A.9)

with  the unit matrix and the matrices  and  as given in (A.11) below and

(A.10)

with

(A.11)

where  and  equal minus the second derivatives of  with respect to  and the mix  and  .

### Appendix II.C Heterogeneity across centers due to clustering

In this appendix we consider the situation in which the centers are clustered by, for example, due to location or type (academic / non-academic medical center). Another example is the covariate hospital size in the nurse-data set, where the clusters are: small, medium, large. Within a hospital/center, all nurses are in the same cluster and have the same covariate value, so that the covariate can not be included in the local regression model as it is collinear with the intercept.

In the calculations of the BFI estimators, we assume that the covariates are independent between the individuals within and across the centers. We, moreover, assume that the outcome variables given the covariates and the cluster level are independent. Suppose that the vector of model parameters in center  is equal to  , where, as before,  is the parameter vector that specifies the distribution of the covariates. The parameter  is the vector of regression parameters which are assumed to be equal in all centers, but excluding the intercept which may vary across the centers. The parameter  for  and with  is the intercept of the model in center  . So,  (with a comma in the subscript) is the parameter in center  , whereas  (without a comma in the subscript) is the parameter for the *k*th category of the center-specific covariate. If  ,  for  and we are in the situation of Appendix II.B, where every center has its own specific intercept value. If  , there are centers  and  with  with  . In the example, the covariate “hospital size” has three levels (small, medium, large). That means that  and the three parameters represent the three intercepts for the three classes of centers. The parameter vector in the (fictive) combined data set  is defined as  .

For simplicity of notation we assume (again) that  and  are independent:  for the combined data set, and locally  in data subset  . For the combined data  we assume a Gaussian prior with mean zero and inverse covariance matrices  for  , and a zero mean Gaussian prior with inverse variance  for  . Also for center  zero mean Gaussian priors are chosen, but with inverse covariance matrix  and inverse variance  . Similar notation and calculations as in Appendix II.B lead to the equation below, instead of the equation (A.8):

with  representing a term that does not depend on  . Let  denote the category of center  for the center-specific covariate. So  . Differentiating  with respect to  and  and setting the derivatives equal to zero, yields the BFI estimators:

with  ,  and  as given in (A.12) below and the estimator  is given by

with minus the second derivatives of  equal to

(A.12)

and 0 for the remaining terms.

## Appendix III Asymptotic theory of the BFI and WAV estimators

In this appendix we compute the asymptotic distribution of the BFI and WAV estimators under the assumption of homogeneity and heterogeneity. In the calculations we assume that the number of clusters *L* is fixed, but the sample sizes within the clusters,  , increase to infinity such that, for  , the fraction  , with  . In all cases we assume no model-misspecification and the independence assumptions stated in Appendix II. This Appendix consists of

- Appendix III.A: Asymptotic distribution of the MAP estimator based on the combined data.
- Appendix III.B: Asymptotic distribution of the BFI and WAV estimators in a homogeneous setting.
- Appendix III.C: Asymptotic distribution of the BFI and WAV estimators in a heterogeneous setting.

### Appendix III.A Asymptotic distribution of the MAP estimator based on the combined data

In this section we study the asymptotic distribution of the MAP estimator  for  in the combined data set. The asymptotic distribution for the MAP estimator for  can be derived similarly.

From literature (Bernstein–Von Mises Theorem^28^) it is known that the MAP estimator is asymptotically Gaussian:

where “  ” means convergence in distribution for the sample size to infinity. Further,  is a vector of zeroes, and  is the inverse Fisher information matrix for the combined populations from all centers. The Fisher information matrix and its inverse have the form

with  and where  and  equal the expectation of the second derivatives of  with respect to  and  , evaluated at the true value of  . The matrix  equals the expectation of the derivative of  with respect to  and  , evaluated at the true value of  .

In the following we rewrite the four submatrices of  in terms of the Fisher information matrices in the local centers. In the next appendices it will be proven that the asymptotic covariance matrices of the BFI-estimators equal these expressions and the BFI estimators are therefore asymptotically efficient.

The data from the different centers are independent. Therefore, the Fisher information matrix can be written as a weighted sum of the Fisher information matrices in the different centers. By the law of large numbers

with  the Fisher information matrix for  in center  and  if  . So  .

In the homogeneous setting all parameters are included in  (there is no parameter  ). Then, for  the Fisher information matrix for  in center  , it follows that  and

(A.13)

The heterogeneous setting is more complex. Suppose the parameter  is assumed to be same across all centers, but  is a vector with center-specific parameters (the index refers to the center). The log likelihood function for center  is a function of  , but not of  with  . Therefore, for the Fisher information matrix  for  in center  , the columns and rows that are related to  contain zeroes only. The matrix  is the Fisher information matrix for  in center  (so not of  like  ), with the blocks  and  , defined in a similar way as in  . Since  is the same across the centers,  . However,  , since  is the Fisher information matrix for  in center  , whereas  is the Fisher information matrix for  in center  ; the dimensions of the matrices are different.

Since the parameter  is a vector with center-specific parameters, the matrix  has a block diagonal matrix with center-specific blocks. Because of this form, it follows that

Since  (the parameter vector  is shared across all centers),

and the asymptotic covariance matrix for  is equal to

(A.14)

The asymptotic covariance matrix for the MAP estimator  equals:

(A.15)

For parameter  the asymptotic covariance matrix equals the  th diagonal block of this matrix. The corresponding block of the matrix  equals  . By the structure of  and the equation (A.14) it follows that  diagonal block of the matrix in (A.15) is given by

(A.16)

### Appendix III.B Asymptotic distribution in homogeneous setting

#### Asymptotic distribution BFI estimator

In Appendix II.A we have derived an expression for the BFI estimator in the homogeneous setting. In this setting, all parameters are included in the vector  ; there is no vector  . The BFI estimator  is defined as

Below, we derive the asymptotic distribution of  :

Because the term

it holds that

Asymptotically, the MAP estimator and the maximum likelihood estimator are equivalent. It follows that the MAP estimator in center  ,  , is asymptotically normal^28^ ^,^ ^29^:  for  the Fisher information matrix in center  . Remember that  is defined as the second derivative of  evaluated at  . If this second derivative is sufficiently smooth near  , it follow by the law of large numbers, that  converges in probability to  .^28^ ^,^ ^29^ By Slutsky’s lemma, it follows that, for every center

Since the data across the *L* centers are assumed to be independent, it follows that

Further, the term

Combining the results, yields

The asymptotic covariance matrix equals  as defined in (A.13), which equals the asymptotic covariance matrix of the MAP estimator based on the combined data. The BFI estimator is asymptotically efficient; no information is lost if the data from the centers can not be combined. Under homogeneity the matrices  , and because  ,

Further, we have seen that the BFI estimator

converges in probability to  .

If the number of centers *L* increases to infinity as well, but  (i.e., the number of centers is smaller in rate than the total sample size), the asymptotic results remain valid, but the derivation needs to be adjusted slightly.

#### Asymptotic distribution of the WAV and Single center estimators

Suppose the MAP estimator  in center  is used for estimating the parameter  , then we obtain

if  . That means that the single-center estimator is not optimal for estimating  unless  (there is only one center).

For the weighted average estimator  for estimating  , we find

with, in the homogeneous setting

as defined in (A.13). In the homogeneous setting, the weighted average estimator is asymptotically efficient as well.

### Appendix III.C Asymptotic distribution of the BFI estimator in heterogeneous setting

#### Asymptotic distribution BFI estimator

Let  and  be the second derivatives of  with respect to  and  , respectively, and let  be the second derivative with respect to both  and  , all evaluated at the MAP estimator  . If these second derivatives are sufficiently smooth in the neighborhood of  , it follows by the law of large numbers that

with the matrices  , and  for center  .

#### Asymptotic behaviour of the BFI estimator for

In Appendix II.B we computed that, under the assumption that  , the BFI estimator for  is equal to:

(A.17)

Define  . Then,

(A.18)

If the derivatives are sufficiently smooth, it follows by the law of large numbers, continuous mapping theorem, and Slutsky’s lemma,^28^ that  converges in probability to  , which equals the left-upper block of the inverse of the Fisher information matrix  in center  . Now, based on similar calculations as in the homogeneous setting

with the asymptotic covariance matrix equal to the asymptotic covariance matrix of the MAP estimator based on all data, given in Equation (A.14). The BFI estimator  is asymptotically efficient for estimating  .

If  and  equal across the centers:

#### Asymptotic behaviour of the BFI estimator of

Under the assumption that  and  , the BFI estimator for  is given by

Then,

(A.19)

We first leave out the last term and consider the asymptotic behaviour of

As explained before, this term equals

In center  the MAP estimator  is asymptotically normal (Bernstein–Von Mises Theorem^28^):

Define the function  . Then, by the continuous mapping theorem

with  the derivative of the function *g* in  . Straightforward calculations show that

which equals the asymptotic covariance matrix of the Gaussian limit distribution for  if the parameter  is known. Apparently, leaving out the last term in equation (A.19), means that we assume that the BFI estimator for  is (almost) equal to the true value  , and thus that  is (almost) known. The result is interesting; the asymptotic accuracy of the BFI estimator for  is increased, because the parameter  can be estimated more accurately with the BFI estimator (and thus using information from the other centers) compared to the situation in which  is estimated based on data from center  only.

We go back to the expression in (A.19):

The asymptotic distribution can be obtained with the continuous mapping theorem and Slutky’s lemma again. The third and last term depends on data from all centers except from center  . Since the data from the different centers are assumed to be independent, it is sufficient to show that the asymptotic distributions of the sum of the first and second terms and of the third term are asymptotically normal and next add the asymptotic mean (which are zero) and the variances. The asymptotic distribution of the sum of the first and second term can obtained with the continuous mapping theorem, like before. The third term is asymptotically normal:

with

If we sum up the variances of the asymptotic zero mean Gaussian distributions of all terms, we obtain

So,

The BFI estimator follows, asymptotically, the same distribution as the MAP estimator based on the combined data, see Equation (A.16), and is asymptotically efficient. If  the asymptotic distribution of  will not change, because  and  converge to zero.

Like in the homogeneous setting, it can be directly seen that the BFI estimator  converges in probability to  . Similarly, the BFI estimator  converges in probability to  , and  to  .

#### Asymptotic distribution of the WAV and single center estimators

If the parameter  is estimated by the MAP estimator from center  ,  , the asymptotic distribution equals

If the weighted average estimator  is used, the asymptotic distribution equals, like before

Asymptotically, both estimators are not efficient, unless  . In that case the weighted average estimator is asymptotically efficient as well.

If we estimate  by its MAP estimator in the corresponding center, the asymptotic distribution equals:

Since  is center-specific, the WAV estimator equals the single center estimator. The estimator  is based on data from center  only, and does not use any information from the other centers for estimating the parameter  . Using information from the other centers,  is estimated more accurately and improves the estimation of  . That is why the BFI estimator  is more accurate than the weighted average estimator.

The asymptotic distribution of the BFI estimators for clustered data (Appendix II.C) can be derived in a similar way, but this is more complicated due to the complex expressions of these estimators. However, since the BFI estimators in the homogeneous and the heterogeneous case with center-specific parameters have shown to be asymptotically efficient and these settings are special cases of the one with clustered data, it is expected that the BFI estimators for the clustered data are asymptotically efficient as well.
