# Bayesian Federated Inference for regression models based on non-shared medical center data – ERRATUM

**DOI:** 10.1017/rsm.2025.23

**Published:** 2025-04-14

**Authors:** Marianne A. Jonker, Hassan Pazira, Anthony C. C. Coolen

An error was made to an author affiliation during the production of this manuscript.

The author Hassan Pazira was incorrectly affiliated with ‘DCN Donders Institute, Faculty of Science, Radboud University, Nijmegen, Netherlands’ their correct affiliation is ‘Research Institute for Medical Innovation, Science Department IQ Health, Section Biostatistics, Radboud University Medical Center, Nijmegen, Netherlands’.

The manuscript has now been updated online and in the PDF version.

The publisher apologises for this error.
